# Genistein–Aspirin Combination Exerts Cytotoxic and Anti-Migratory Effects in Human Colorectal Cancer Cells

**DOI:** 10.3390/life14050606

**Published:** 2024-05-09

**Authors:** Claudia Iftode, Stela Iurciuc, Iasmina Marcovici, Ioana Macasoi, Dorina Coricovac, Cristina Dehelean, Sorin Ursoniu, Andreea Rusu, Simona Ardelean

**Affiliations:** 1 Faculty of Medicine, “Victor Babeș” University of Medicine and Pharmacy from Timisoara, Eftimie Murgu Square No. 2, 300041 Timisoara, Romania; claudia.geaba@umft.ro (C.I.); sursoniu@umft.ro (S.U.); 2 Faculty of Pharmacy, “Victor Babes” University of Medicine and Pharmacy from Timisoara, Eftimie Murgu Square No. 2, 300041 Timisoara, Romania; iasmina.marcovici@umft.ro (I.M.); macasoi.ioana@umft.ro (I.M.); dorinacoricovac@umft.ro (D.C.); cadehelean@umft.ro (C.D.); 3 Research Center for Pharmaco-Toxicological Evaluations, Faculty of Pharmacy, “Victor Babes” University of Medicine and Pharmacy from Timisoara, Eftimie Murgu Square No. 2, 300041 Timisoara, Romania; 4 Center for Translational Research and Systems Medicine, “Victor Babes” University of Medicine and Pharmacy from Timisoara, Eftimie Murgu Square No. 2, 300041 Timisoara, Romania; 5 Faculty of Pharmacy, Vasile Goldis Western University of Arad, Revolutiei Bvd 94, 310130 Arad, Romania; rusu.andreea@uvvg.ro (A.R.); simonaaardelean@yahoo.com (S.A.)

**Keywords:** genistein, aspirin, colorectal cancer, cytotoxicity, angiogenesis, chemoprevention, migration

## Abstract

Colorectal cancer (CRC) is a heterogenous pathology with high incidence and mortality rates globally, but it is also preventable so finding the most promising candidates (natural compounds or repurposed drugs) to be chemopreventive alternatives has become a topic of interest in recent years. The present work aims to elucidate the potential effects of a combination between genistein (GEN), an isoflavone of natural origin, and aspirin (ASA) in CRC prevention/treatment by performing an in vitro evaluation in human colorectal cancer cells (HCT-116) and an in ovo analysis using the chick embryo chorioallantoic membrane (CAM) model. Cell viability was verified by an MTT (migratory potential by scratch) assay, and the expressions of MMP-2 and MMP-9 were analyzed using RT-qPCR. Our results indicated a dose-dependent cytotoxic effect of ASA (2.5 mM) + GEN (10–75 µM) combination characterized by reduced cell viability and morphological changes (actin skeleton reorganization and nuclei deterioration), an inhibition of HCT-116 cells’ migratory potential by down-regulating MMP-2 and MMP-9 mRNA expressions, and an antiangiogenic effect by modifying the vascular network. These promising results raise the possibility of future in-depth investigations regarding the chemopreventive/therapeutical potential of ASA+GEN combination.

## 1. Introduction

Colorectal cancer (CRC) is a complex pathogenesis dependent on both patient-related (non-modifiable—i.e., age, genetic predisposition, and inflammatory bowel illness) and environmental (modifiable) factors [1,2]. According to the GLOBOCAN 2022 report, in Romania, CRC occupies the first place in both incidence (13,541 new cases) and mortality (7381 deaths) in both sexes, and the second place as the most frequently diagnosed in females after breast cancer [3]. CRC is considered to have a slow progression, but late diagnosis places this type of cancer as the second cause of cancer-associated mortality and the third in incidence globally [1,2]. Similar to other types of cancer, recent studies endorse the sexual-related disparities in CRC incidence rates among men and women. A higher CRC incidence in men is reported worldwide and these differences are associated with sexual dysmorphism (e.g., sex chromosomes, sex hormones, and immunity), but also with the exposome (environmental risk factors), as thoroughly discussed by Baraibar et al. in an excellent review [4]. Moreover, CRC mortality rates are also higher in men compared to women, and women (mainly premenopausal women) have a better overall cancer-specific survival rate [5]. It is still debatable how sex hormones can exhibit antitumor effects in CRC since they act as carcinogenic in other tissues, but based on existent information, this protection can be attributable to the roles that estrogen receptors—ER (mainly ERβ)—play in multiple processes involved in CRC development and progression, such as tumor microenvironment (hypoxia, cellular metabolism, drug metabolism, angiogenesis, gut microbiome, etc.), ion channel function, immunity, apoptosis of cancer cells, and cell cycle regulation [5,6]. In addition, the lack of estrogen in post-menopausal women might increase the risk of developing right-sided CRC tumors that are characterized by microsatellites instability and a poorer prognosis [4].

Gender disparities were also identified in the case of CRC-related environmental factors, as women have healthier dietary habits than men and are oriented towards the consumption of dietary supplements [5,7]. Environmental CRC risk factors such as body weight, diet, alcohol consumption, smoking, and physical activity are controllable and represent key targets for chemopreventive measures [2]. The use of nutritional/dietary supplements (e.g., calcium, vitamin D, folate, phytochemicals, n-3 PUFAs, etc.) or pharmacologically active compounds (re-purposed drugs—e.g., aspirin) are ranked among the most applied CRC prevention strategies [2,8,9]. 

In recent years, an emerging interest towards the use of natural compounds as potential alternatives for CRC management was noted, due to their multiple benefits in different stages of the CRC pathogenesis, as follows: (i) cytotoxic activity against cancer cells after tumor onset, (ii) preventive effect in cancer recurrence or metastasis, and (iii) enhancement of different chemotherapeutics effects after drug resistance settlement [8].

A natural compound that was investigated for beneficial effects in CRC management and that showed promising results is genistein (GEN), an isoflavone and member of the flavonoids family that is found in high amounts in soybeans [10]. A particular feature of GEN is represented by its chemical structure (5,7-dihydroxy-3-(4-hydroxyphenyl) chromen-4-one), which resembles the mammalian estrogen. This natural compound possesses various biological effects; it is antioxidant, anti-inflammatory, antiangiogenic, protective against osteoporosis, decreases the risk of cardiovascular pathology, palliates postmenopausal symptoms, and promotes anticancer activity [10]. With regard to GEN’s impact in CRC, a number of preclinical studies described several mechanisms of action, such as (i) a decrease in cellular proliferation, induction of apoptosis, and suppression of the epidermal growth factor receptor (EGFR), as well as of estrogen receptor 1 (ESR1) expressions in colorectal cancer cells (HCT-116) [11]; (ii) inhibition of HCT-116 cells proliferation, inhibition of apoptosis, down-regulation of Akt, SGK1 and miR-95 mRNA expressions, and suppression of tumor growth in vivo [12]; (iii) inhibition of colon cancer cells’ (HT-29) migration by reversing epithelial-to-mesenchymal transition via suppression of the Notch1/NF-κB/slug/E-cadherin pathway [13]; (iv) induction of apoptosis in colorectal cancer cell lines SW480 and SW620 by stimulating the oxidative reactive species (ROS) production [14]; and (v) suppression of colon cancer cells’ (HT-29) invasion and migration via demethylation and modulation of Wnt signaling pathway [15]. 

Another acknowledged CRC chemoprevention strategy is the use of low-dose aspirin (≤100 mg/day), a non-steroidal anti-inflammatory (NSAID) agent that was recommended in 2016 by the US Preventive Services Task Force (USPSTF) based on the data obtained from cohort studies and randomized clinical trials that applied to individuals aged 50 to 59 years with defined cardiovascular risk and a reduced risk of bleeding [16]. 

Aspirin, also known as acetylsalicylic acid (ASA), is an established therapeutical agent that demonstrated multiple dose-dependent biological effects including analgesic, antipyretic, antirheumatic, and cardiovascular prophylactic agent. In recent years, the anticancer effect of aspirin, as a repurposed drug, was intensively studied and several potential mechanisms of action were described, as follows: (i) suppression of prostaglandin E2 (PGE2) formation via COX-1 (cyclooxygenase 1) inhibition; (ii) reduction in pro-inflammatory cytokines expression via COX-2 inhibition; (iii) induction of apoptosis in cancer cells; (iv) modulation of immune response; and (v) inhibition of proto-oncogenes, etc. [16,17,18]. Even though a significant body of evidence exists regarding the mechanisms involved in the anticancer effect of aspirin, this subject is far from fully elucidated. 

Consistent data support the anticancer/chemopreventive potential of aspirin in different types of cancer, including lung, breast, ovarian, stomach, and colorectal cancer [17,19,20]. Still, an update of the USPSTF recommendation regarding the use of low-dose aspirin as a prophylactic agent in reducing CRC incidence was published in 2022 and they concluded that the evidence was insufficient, and their modeling study assumed no effect of aspirin on CRC incidence [21]. Hence, the chemopreventive activity of aspirin remains debatable and further studies are required to shed some light in this direction. 

In recent years, the potential synergistic effect (as chemopreventive alternatives for CRC) of different anticancer drugs and natural compounds, including 5-fluorouracil (5-FU) and genistein, was investigated, as was the association of aspirin with dietary plants, and the results were promising [22,23]. Moreover, in a recent study, several hybrid scaffolds based on aspirin and genistein were analyzed in terms of their chemopreventive potential in colorectal cancer cells, and a synergistic effect was recorded, but further studies are required to explain the protective effect of aspirin–genistein associations [24].

Taking into consideration the data presented above and the possibility that women (the post-menopausal women, in particular) might associate an nutraceutical based on GEN (which act as an ER agonist) and ASA – as chemoprevention for CRC, the present study investigated the effects of an ASA+GEN combination in human colorectal cancer cells (HCT-116) by performing: (i) an in vitro evaluation of cell viability, cell morphology changes, and migratory and invasion potential, and (ii) an in ovo analysis to determine the antiangiogenic activity.

## 2. Materials and Methods

### 2.1. Materials

The active substances tested were aspirin (ASA) and genistein (GEN), and together with the specific reagents applied, including trypsin-EDTA (ethylenediaminetetraacetic acid) solution, DMSO (dimethyl sulfoxide), PBS (phosphate buffer saline), FCS (fetal calf serum), the antibiotic combination (penicillin–streptomycin), and DAPI (4′,6-diamidino-2-phenylindole, dihydrochloride), along with the Triton X-100 and MTT viability kit, were delivered by Sigma Aldrich, Merck KgaA (Darmstadt, Germany). The specific media used for the growth of the monolayer cell cultures, DMEM (Dulbecco’s minimal essential medium; 30-2002™) and McCoy’s 5A medium (30-2007™), were bought from ATCC (American Type Cell Collection, Lomianki, Poland). The nuclear labeling kit Hoechst 33342 was ordered from Invitrogen (Carlsbad, CA, USA) and Texas Red™-X Phalloidin was purchased from Thermo Fisher Scientific Inc. (Waltham, MA, USA). All the reagents and kits used in the present study are certified and comply with the standards of use in experiments carried out in biological environments on cell cultures and were applied according to the recommendations of the manufacturing companies.

### 2.2. Cell Culture Conditions

The cell lines used in the research conducted, human keratinocytes (HaCaT, 300493) and human colorectal carcinoma cells (HCT-116, CRL-1619™), were received from CLS (Cell Lines Service GmbH, Eppelheim, Germany) and ATCC, respectively.

The cells were cultured following the manufacturers’ recommendations, as follows: HaCaT cells were grown in a specific medium, DMEM supplemented with 10% FBS, 4.5 g/L Glucose, 4 mM L-Glutamine, 1.5 g/L sodium bicarbonate, and 1.0 mM sodium pyruvate, and HCT-116 cells required McCoy 5A specific medium supplemented with 10% FBS. The trypsin/EDTA (0.05%/0.025%—for HaCaT cells and 0.25%—for HCT-116 cells, respectively) solution and penicillin–streptomycin antibiotic mixture (100 U/mL—100 µg/mL) were also used, and the cells were cultured in standard 37 °C and 5% CO_2_ conditions in an incubator dedicated to cell cultures.

### 2.3. Cell Viability Assay

The assessment of cells’ viability (HaCaT and HCT-116) following stimulation with the compounds of interest (ASA, GEN, and the ASA+GEN combination) was performed using the MTT test after a 72-h interval. Briefly, the experimental evaluation involved the cultivation of cells in 96-well plates, stimulation with ASA (1, 2.5, 5 and 7.5 mM), GEN (10, 25, 50 and 75 µM), and ASA (2.5 mM) + GEN (10, 25, 50 and 75 µM), and incubation for 72 h, at a temperature of 37 °C and 5% CO_2_, along with the addition of a fresh culture medium (100 µL) and MTT reagent (10 µL), incubation for 3 h in conditions similar to cell growth, and the addition of the solubilizing solution (100 µL) followed by the plates’ incubation at room temperature for half an hour. In the final step, the absorbance was read at two different wavelengths (570 nm and 630 nm) using Cytation 5 (BioTek Instruments Inc., Winooski, VT, USA), a multimodal plate reader. 

### 2.4. Cellular Morphology Assessment

The impact of the compounds of interest (ASA, GEN, and the ASA+GEN combination) on cell morphology and confluency (HCT-116) was verified by microscopic evaluation. In brief, cell seeding was performed in 12-well plates, the images were taken under bright field illumination, and the analysis period was 72 h after stimulation with different concentrations of active substances. The equipment used was the Cytation 1 microscope (BioTek Instruments Inc., Winooski, VT, USA) and image processing was performed with the help of Gen5™ software Version 3.14 (Microplate Data Collection and Analysis Software).

### 2.5. Immunofluorescence Staining of Cellular Components

Cells’ nuclei and actin filaments (cellular components of interest) were investigated through immunofluorescence staining. Human colorectal carcinoma cells (HCT-116) were seeded to 12-well plates, stimulated with ASA (1, 2.5, 5 and 7.5 mM), GEN (10, 25, 50 and 75 µM), and the ASA (2.5 mM) + GEN (10, 25, 50 and 75 µM) combination. After 72 h, the cells were subjected to staining steps, as follows: (i) washing with ice-cold PBS, (ii) fixation with 4% paraformaldehyde solution in PBS, (iii) treatment with 0.1% Triton X/PBS, and (iv) blocking with 30% FBS in 0.01% Triton X. The immunofluorescent markers used were DAPI for nuclei and Texas Red™-X Phalloidin for actin filaments, following the protocols provided by the manufacturers. Images of interest were captured using Cytation 1 and processed and analyzed with Gen5™ software. The quantification of the nuclei presenting apoptosis-specific features was performed by calculating the apoptotic index (%) in DAPI-stained cells according to the following formula:

(1) $$\mathrm{Apoptotic}\;\mathrm{index}\;\left(\%\right)=\frac{\mathrm{Number}\;\mathrm{of}\;\mathrm{apoptotic}\;\mathrm{nuclei}}{\mathrm{Total}\;\mathrm{number}\;\mathrm{of}\;\mathrm{nuclei}}\;\times \;100$$

### 2.6. In Vitro Migration Assay

The effect on cell migration (HCT-116 cells) following stimulation with ASA (2.5 mM), GEN (10 and 25 µM), and ASA (2.5 mM) + GEN (10 and 25 µM) was analyzed through the wound healing method (scratch assay). Briefly, 100,000 cells/mL/well were cultured in 12-well plates, a manual scratch was performed with a pipette tip in the middle of each well, and the cells were stimulated with compounds of interest for 24 h. Representative images were taken at the initial time of 0 h and at the final time of 24 h using an inverted microscope (Olympus IX73) equipped with a DP74 camera which were bought from Olympus (Tokyo, Japan). The measurement of the width of the wounds (correlated with the regeneration effect) was made after 24 h with CellSense Dimension 1.17 (Olympus, Tokyo, Japan). The calculation of the wound healing rate, the effective quantification of cell migration, was carried out using the formula published in the literature [25]. 

### 2.7. RT-qPCR Analysis of MMP-2 and MMP-9 Markers Expression

For the RT-qPCR analysis, HCT-116 cells were cultured in 6-well plates and treated with ASA (2.5 mM), GEN (25 µM), and ASA (2.5 mM) + GEN (25 µM) for 24 h. The RNA extraction was performed using the Invitrogen™ PureLink™ RNA Mini Kit, and its concentration in each sample was evaluated with the help of the DS-11 spectrophotometer (DeNovix, Wilmington, DE, USA). The bioconversion of the extracted RNA to cDNA was realized with the Power SYBR™ Green RNA-to-C_T_™ 1-Step Kit (Thermo Fisher Scientific, Inc., Waltham, MA, USA). The quantitative real-time PCR protocol was conducted utilizing the Quant Studio 5 real-time PCR system (Thermo Fisher Scientific, Inc., Waltham, MA, USA) at the following thermal conditions: 48 °C for 15 min, 95 °C for 10 min, 95 °C for 15 min (40 cycles), and 60 °C for 1 min. The primer pairs used are presented below (Table 1).

### 2.8. Chorioallantoic Membrane Assay (CAM)

For the evaluation of the test compounds’ effect on angiogenesis, the chick embryo chorioallantoic membrane (CAM) was used as a biological model. The following test substances were applied to the CAM: ASA—2.5 mM, GEN—50 µM, and the combination of both (ASA 2.5 mM + GEN 50 µM). For this purpose, the eggs were prepared according to the subsequent steps: (i) on the first day, the eggs were cleaned and placed in an incubator at constant humidity (45%) and temperature (37.5 °C); (ii) on the third day of embryonic development (EDD3), a small hole was made at the tips of the eggs, from which approximately 5–7 mL of albumen was extracted; and (iii) on the fourth day of embryonic development (EDD4), a window was created at the top of the egg, then covered with adhesive tape, and the eggs were returned to the incubator until the start of the experiment.

The evaluation of test compounds’ impact on angiogenesis commenced on the sixth day of embryonic development (EDD6) and spanned over five days. Simply, a plastic ring was applied to the CAM, within which 10 µL of test substances were administered daily. Angiogenesis assessment was conducted by daily photography of the vascular plexus utilizing a stereomicroscope (Discovery v.8), a color camera (Zeiss Axiocam 105), and software (ZEN core Version 3.8) bought from Zeiss (Göttingen, Germany). To quantitatively assess the anti-angiogenic effects of ASA, GEN, and ASA+GEN in comparison to the control, a total area of 8 × 10^6^ square pixels surrounding the region separated by the silicon ring was chosen. The IKOSA Prism Application CAM assay (v3.1.0) was then utilized to perform quantitative evaluations of vessel numbers, branching points, and total vessel area.

### 2.9. Statistical Analysis

All data were described as means ± standard deviation (SD) obtained after performing three experiments carried out in triplicate. The statistical difference resulted from the application of the specific post-tests of GraphPad Prism software version 9.2.0 for Windows (GraphPad Software, San Diego, CA, USA). Differences between control cells (non-stimulated cells) and stimulated cells that showed statistical significance are highlighted with *, which represents the following: * *p* < 0.05; ** *p* < 0.01; *** *p* < 0.001; **** *p* < 0.0001. 

## 3. Results

### 3.1. ASA+GEN Combination Triggers a Dose-Dependent Decline in HCT-116 Cells’ Viability

**Figure 1:**
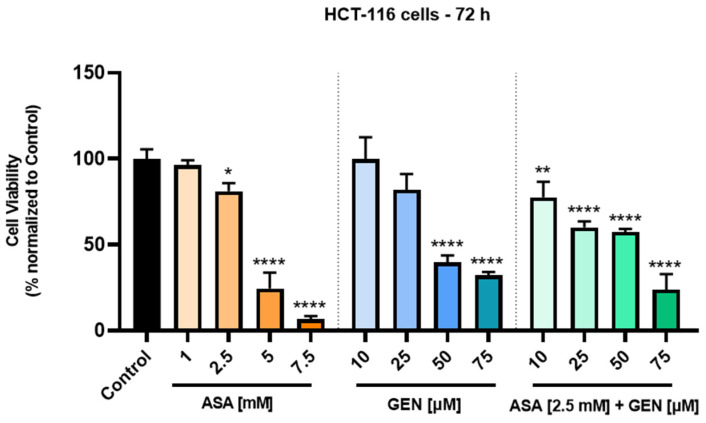
Assessment of the viability of human colorectal cancer cells—HCT-116—following a 72 h treatment with aspirin (ASA—1, 2.5, 5, and 7.5 mM) and genistein (GEN—10, 25, 50, and 75 µM), as well as with an aspirin (ASA—2.5 mM) + genistein (GEN—10, 25, 50, and 75 µM) combination by MTT assay. The results are presented as cell viability percentage (%) normalized to untreated (control) cells. The data represent the mean values ± standard deviation (SD) (N = 3, triplicate). One-way ANOVA analysis followed by Dunnett’s multiple comparisons post-test was applied to determine the statistical differences in rapport with control (* *p* < 0.05, ** *p* < 0.01, and **** *p* < 0.0001).

To investigate the potential cytotoxic effect of the ASA (2.5 mM) + GEN (10–75 µM) combination on human colorectal cancer cells’ (HCT-116) viability, an MTT assay was conducted after a 72 h incubation period. Simultaneously, the effect of each test compound (ASA and GEN) was tested by applying the same experimental conditions. The results indicated a dose-dependent decrease in the percentage of HCT-116 viable cells compared to control cells (untreated) after both ASA and GEN treatment (Figure 1). ASA showed a significant cytotoxicity in HCT-116 cells, at concentrations higher than 1 mM, by reducing the cells’ viability to 81.05% (at 2.5 mM), 24.44% (at 5 mM), and 6.62% (at 7.5 mM). In the case of GEN, cytotoxicity was noticed only at the highest tested concentrations (50 µM—39.6%, and 75 µM—32.2%). 

For the assessment of the ASA+GEN combination effect, the lowest concentration of ASA that reduced HCT-116 cells’ viability was selected—2.5 mM—along with all the concentrations tested for GEN (10, 25, 50, and 75 µM). The MTT assay performed demonstrated a significant dose-dependent decrease in the viability percentage of ASA+GEN-treated cells, with their decrease being more pronounced than in the case of GEN-treated ones, except for the concentration of 50 µM. However, compared to the individual treatments, the association of ASA with GEN induced significant reductions in the viability of HCT-116 cells at all tested concentrations. The obtained values were as follows: ASA 2.5 mM + GEN 10 µM—77.6%, ASA 2.5 mM + GEN 25 µM—59.9%, ASA 2.5 mM + GEN 50 µM—57.3%, and ASA 2.5 mM + GEN 75 µM—23.8%. 

**Figure 2:**
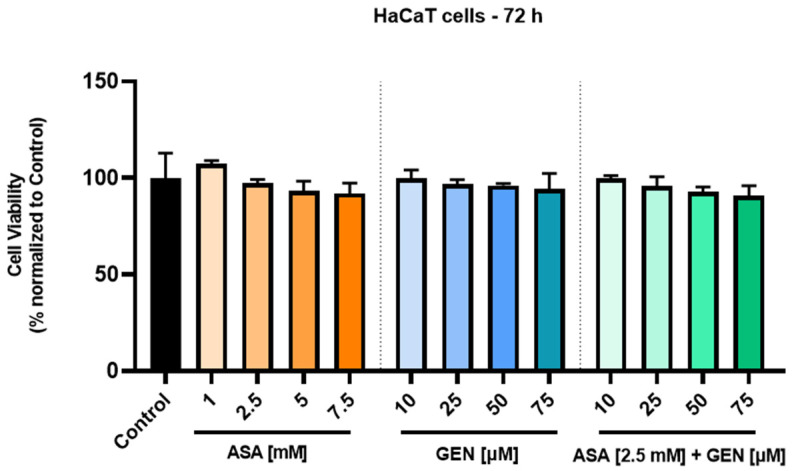
Assessment of the viability of human keratinocytes—HaCaT—following a 72 h treatment with aspirin (ASA—1, 2.5, 5 and 7.5 mM) and genistein (GEN—10, 25, 50 and 75 µM), as well as with an aspirin (ASA—2.5 mM) + genistein (GEN—10, 25, 50 and 75 µM) combination by MTT assay. The results are presented as cell viability percentage (%) normalized to control (untreated) cells. The data represent the mean values ± standard deviation (SD) (N = 3, triplicate). One-way ANOVA analysis followed by Dunnett’s multiple comparisons post-test was applied to determine the statistical differences in rapport with control.

The cell viability of healthy human keratinocytes (HaCaT) was also evaluated to assess possible cytotoxic effects of the test compounds (ASA, GEN) and their combination (ASA+GEN), as shown in Figure 2. The effect exerted on healthy cells proved to be dose-dependent in all three cases, without significant influences. In ASA-treated cells, a slight stimulatory effect was recorded at the lowest concentration (1 mM, 107.23%), while at the highest concentration (7.5 mM) a mild decrease in viable cells (91.88%) was noted. In the case of GEN-treated cells, at the lowest concentration (10 μM), viability was not affected (99.72%), while at the highest concentration (75 μM), a slight decrease in viable cells (94.31%) was observed. The ASA (2.5 mM) + GEN (10–75 μM) combination induced similar effects to those exerted by the parent compounds, as follows: at the lowest concentration (ASA 2.5 mM + GEN 10 μM), no toxicity on keratinocytes was recorded, whereas ASA (2.5 mM) + GEN (75 μM) produced a slight decrease in the number of viable cells (90.64%).

### 3.2. ASA+GEN Combination Induces Apoptotic-like Morphological Changes in HCT-116 Cells

**Figure 3:**
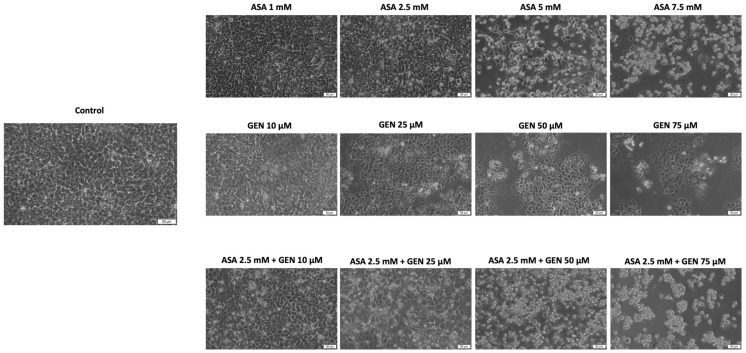
Effect of aspirin (ASA—1, 2.5, 5 and 7.5 mM), genistein (GEN—10, 25, 50 and 75 µM), and the aspirin (ASA—2.5 mM) + genistein (GEN—10, 25, 50 and 75 µM) combination on HCT-116 cells’ morphology following a 72 h treatment. Representative images of the cells’ appearance after 72 h (scale bars 50 µm).

Furthermore, the impact of ASA (1–7.5 mM), GEN (10–75 µM), and the ASA (2.5 mM) + GEN (10–75 µM) combination on cellular morphology in HCT-116 cells it was evaluated through microscopic inspection, with modifications/alterations of cells’ shape and morphology being hallmarks of cellular injury, including cell death (apoptosis and necrosis). As can be noticed in Figure 3, control cells (untreated) show an epithelial morphology and are strongly attached and highly confluent. The ASA (1–7.5 mM) treatment, starting with the 2.5 mM concentration, induced the occurrence of small, round/spherical, and detached cells floating in the culture medium, and their number became significantly higher at the highest concentration tested (7.5 mM), with their confluence also being affected. A similar effect was observed after GEN treatment (10–75 µM). In the case of the ASA+GEN treatment, the presence of small, round, and floating cells can be noticed even in the lowest concentration tested wells (ASA 2.5 mM + GEN 10 µM); the higher concentrations (ASA 2.5 mM + GEN 50, 75 µM) induced a considerable elevation in the number of apoptotic-like cells and a reduction in cells’ confluency, observations that support the data presented in the cell viability section.

### 3.3. ASA+GEN Combination Determines Actin Filaments’ Reorganization and Nuclear Changes in HCT-116 Cells

**Figure 4:**
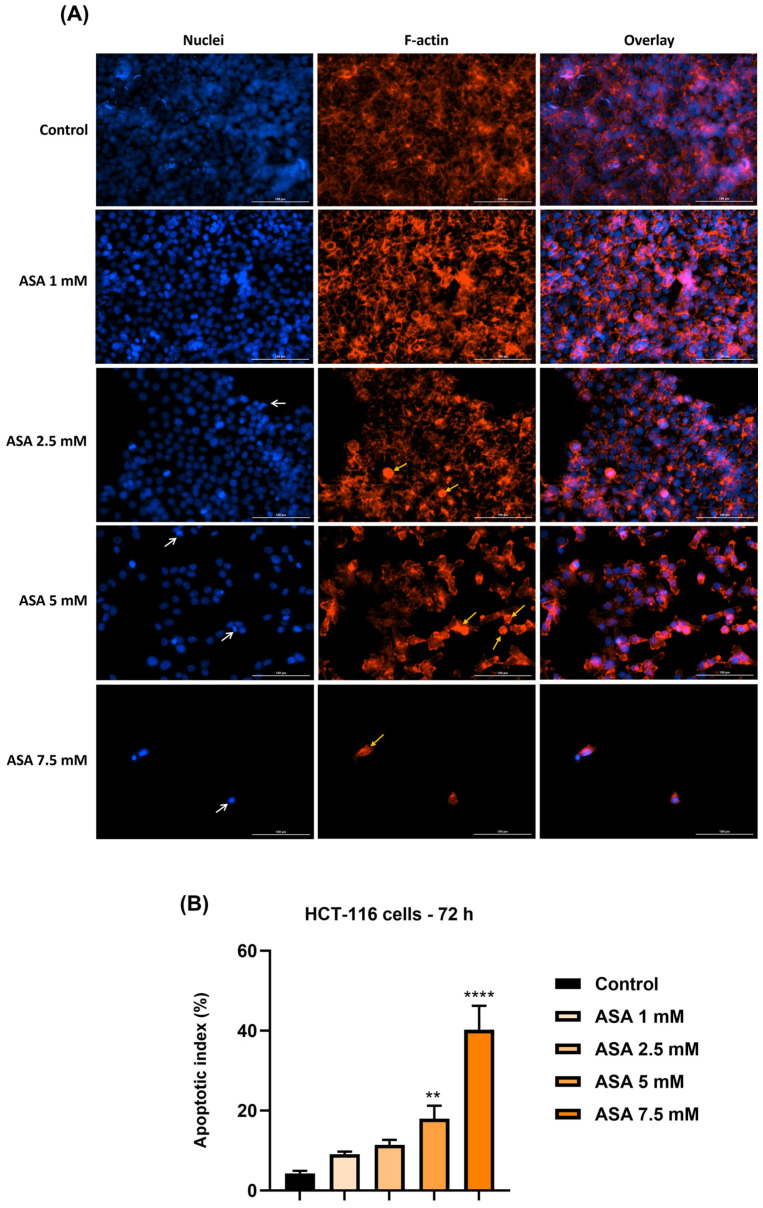
(**A**) Visualization by immunofluorescence staining of aspirin (ASA—1, 2.5, 5, and 7.5 mM) treatment impact on nuclei (blue) and actin filaments (red) in HCT-116 cells at 72 h post-treatment. White arrows indicate nuclear modifications and yellow arrows show actin cytoskeleton remodeling. Scale bars were 100 µm. (**B**) Apoptotic index (%) calculation in DAPI-stained HCT-116 cells treated with aspirin (ASA—1, 2.5, 5, and 7.5 mM) for 72 h. The data represent the mean values ± standard deviation (SD) (N = 3, triplicate). One-way ANOVA analysis followed by Dunnett’s multiple comparisons post-test was applied to determine the statistical differences in rapport with control (** *p* < 0.01 and **** *p* < 0.0001).

**Figure 5:**
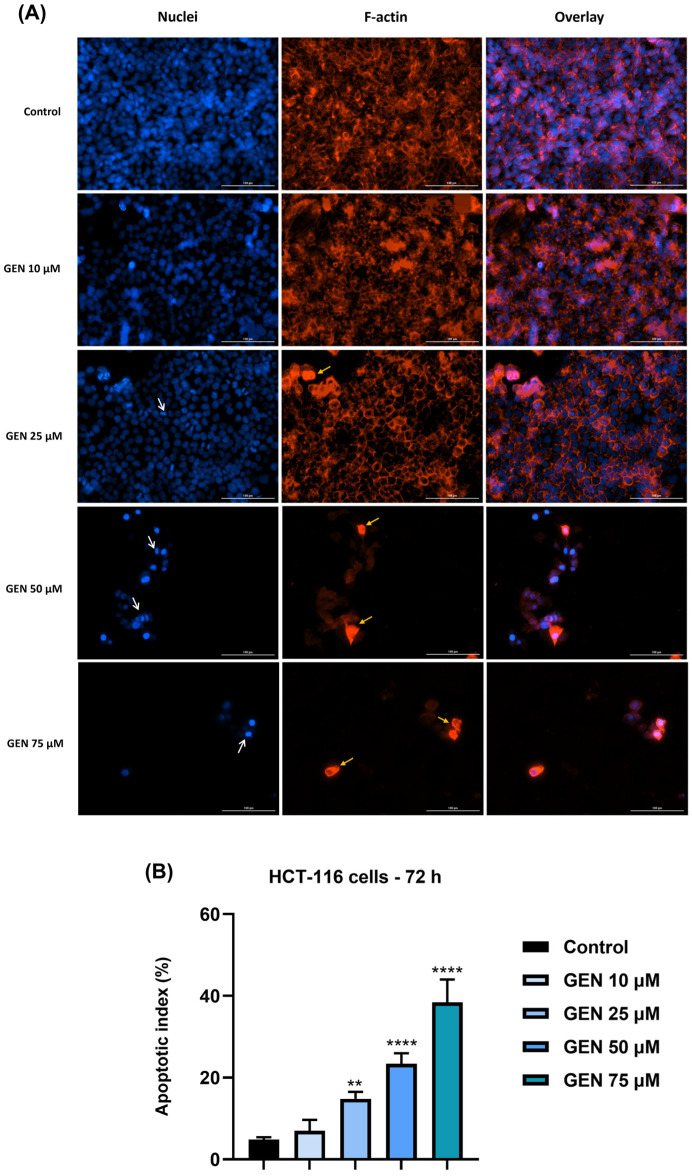
(**A**) Visualization by immunofluorescence staining of genistein (GEN—10, 25, 50, and 75 µM) treatment impact on nuclei (blue) and actin filaments (red) in HCT-116 cells at 72 h post-treatment. White arrows indicate nuclear modifications and yellow arrows show actin cytoskeleton remodeling. Scale bars were 100 µm. (**B**) Apoptotic index (%) calculation in DAPI-stained HCT-116 cells treated with genistein (GEN—10, 25, 50, and 75 µM) for 72 h. The data represent the mean values ± standard deviation (SD) (N = 3, triplicate). One-way ANOVA analysis followed by Dunnett’s multiple comparisons post-test was applied to determine the statistical differences in rapport with control (** *p* < 0.01, and **** *p* < 0.0001).

**Figure 6:**
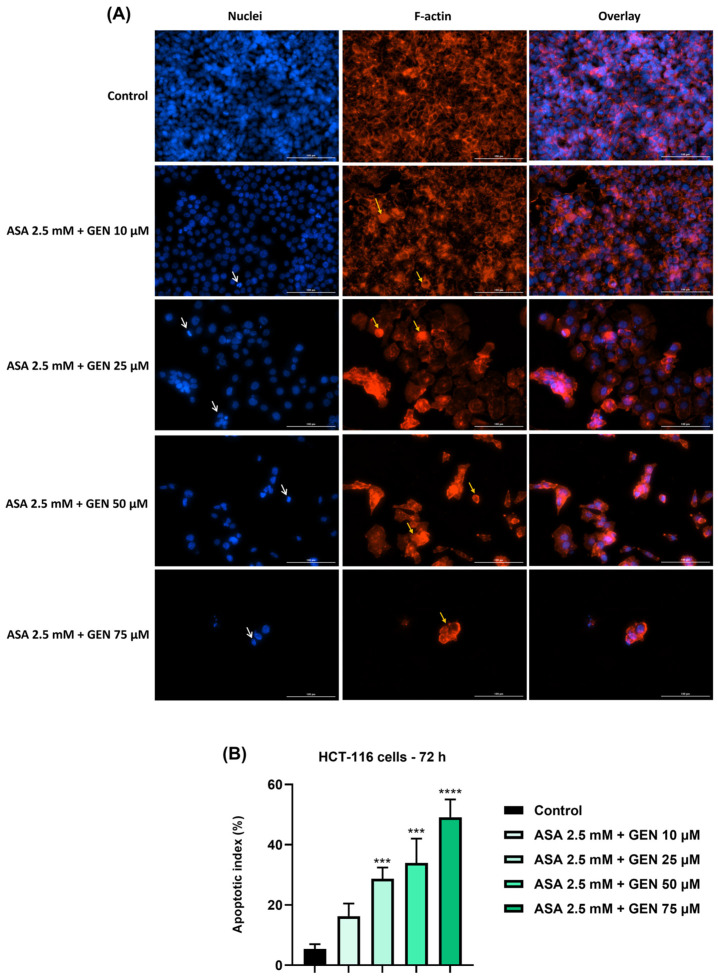
(**A**) Visualization by immunofluorescence staining of the aspirin (ASA 2.5 mM) + genistein (GEN—10, 25, 50, and 75 µM) combination treatment impact nuclei (blue) and actin filaments (red) in HCT-116 cells at 72 h post-treatment. White arrows indicate nuclear modifications and yellow arrows show actin cytoskeleton remodeling. Scale bars were 100 µm. (**B**) Apoptotic index (%) calculation in DAPI-stained HCT-116 cells treated with aspirin (ASA 2.5 mM) + genistein (GEN—10, 25, 50, and 75 µM) for 72 h. The data represent the mean values ± standard deviation (SD) (N = 3, triplicate). One-way ANOVA analysis followed by Dunnett’s multiple comparisons post-test was applied to establish the statistical differences in rapport with control (*** *p* < 0.001, and **** *p* < 0.0001).

To confirm the changes observed after the cellular morphology evaluation of HCT-116 cells by microscopic evaluation following the treatment of test compounds (ASA, GEN, and ASA+GEN combination) for 72 h, immunostaining assays were conducted to visualize the actin cytoskeletal filaments and the nuclear aspect, as well as to analyze the percentage of apoptosis-specific nuclei (Figure 4, Figure 5 and Figure 6). As can be seen in the control cells (untreated), the actin filaments are dispersed throughout the cytoplasm. No marked changes in actin filaments were noticed in the group of cells treated with 1 mM ASA, whereas by increasing the concentration of ASA (2.5, 5 and 7.5 mM), significant changes were recorded, such as the presence of round cells, densely stained, and a reduction in actin filament mass; these modifications were dose dependent. The nuclear staining using DAPI indicated round nuclei with no signs of chromatin condensation or nuclear shrinkage in control cells and the cells treated with 1 mM ASA (Figure 4). Nuclear modifications such as nuclear shrinkage, chromatin condensation, and blebbing were detected in the HCT-116 cells treated with higher concentrations of ASA (2.5, 5, and 7.5 mM), data that support the cell viability results. Compared to the control cells, an increase in the percentage of apoptotic nuclei (AI) was observed in HCT-116 cells treated with ASA at all concentrations; however, statistical significance was reached only for ASA 5 and 7.5 mM.

Similar to the effects of the ASA treatment, as described above, GEN treatment (25, 50, and 75 µM) for 72 h (Figure 5) induced changes characterized by a reorganization of actin filaments, round-shaped cells intensely stained, nuclear shrinkage, and chromatin condensation. At the lowest concentration tested for GEN (10 µM), no apparent changes were detected. The 72 h treatment of the cells with GEN also increased the number of the nuclei presenting morphological features similar to an apoptotic cell death, with a significant elevation in AI being noted for the highest tested concentrations (25–75 µM).

We then evaluated the impact of the ASA (2.5 mM) + GEN (10, 25, 50 and 75 µM) combination on actin filaments and nuclei in HCT-116 cells (Figure 6). The results obtained indicated a significant dose-dependent effect at both the cellular and nuclear level, as follows: reorganization of actin filaments distribution (more condensed on the edges of the cells), modification of cells’ shape (spherical cells densely stained), nuclear shrinkage, chromatin condensation, and nuclear fragmentation. The most significant changes were noticed at the highest concentrations tested (ASA 2.5 mM + GEN 50 and 75 µM). The results described above highlight that the ASA+GEN combination has a cytotoxic effect in colorectal cancer cells (HCT-116). The highest increases in AI were registered after the HCT-116 cells’ treatment with ASA 2.5 and GEN 25, 50, and 75 µM.

### 3.4. ASA+GEN Combination Inhibits HCT-116 Cells’ Migratory Potential

**Figure 7:**
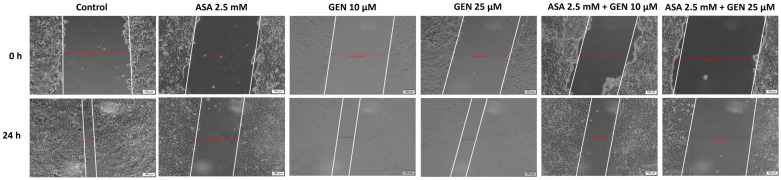
Effect of aspirin (ASA—2.5 mM), genistein (GEN—10 and 25 µM), and the aspirin (ASA—2.5 mM) + genistein (GEN—10 and 25 µM) combination on HCT-116 cells’ migratory potential. Representative images of the appearance of the scratch/gap after 24 h (scale bars—100 µm).

**Figure 8:**
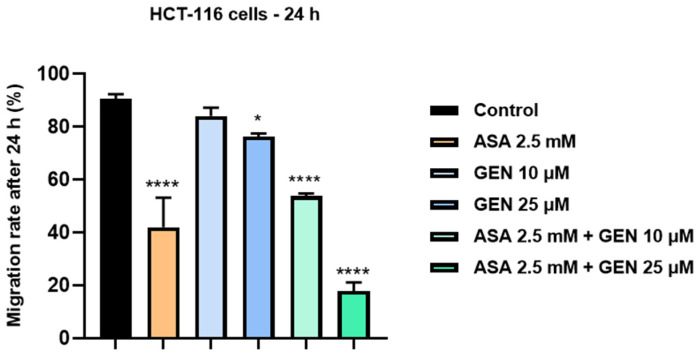
Effect of aspirin (ASA—2.5 mM), genistein (GEN—10 and 25 µM), and the aspirin (ASA −2.5 mM) + genistein (GEN—10 and 25 µM) combination on HCT-116 cells’ migratory potential. Graphic representations of the percentage (%) of scratch/gap coverage after 24 h. The statistical differences between the untreated (control) and the treated (stimulated) group were calculated by one-way ANOVA analysis followed by Dunnett’s multiple comparisons post-test (* *p* < 0.05 and **** *p* < 0.0001).

Since migratory ability represents a key function for cancer cells, we decided to pursue the impact of the test compounds on HCT-116 cells’ migratory potential (Figure 7 and Figure 8). To achieve this goal, a gap closure assay (scratch assay) was performed for a 24 h period, and only the sub-toxic concentrations of test compounds (ASA 2.5 mM, GEN 10 and 25 µM, and ASA 2.5 mM + GEN 10, 25 µM) were verified. A significant inhibition of HCT-116 cells’ migratory ability was obtained after a 24 h exposure to ASA 2.5 mM compared to control cells (untreated). In the case of GEN (10, 25 µM), a slight inhibitory effect was noticed compared to control cells, whereas the ASA (2.5 mM) + GEN (10, 25 µM) combination proved a strong inhibitory effect on cells’ migration. 

### 3.5. ASA+GEN Combination Inhibits MMP-2 and MMP-9 Expressions in HCT-116 Cells

**Figure 9:**
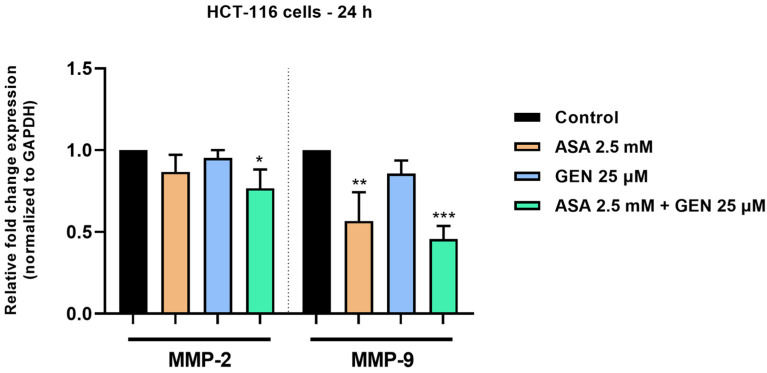
Effect of aspirin (ASA—2.5 mM), genistein (GEN—25 µM), and the aspirin (ASA −2.5 mM) + genistein (GEN—25 µM) combination on the relative fold change expression of metalloproteinases -2 and -9 (MMP-2, and MMP-9) in HCT-116 cells after 24 h of treatment. The expressions were normalized to GAPDH used as housekeeping gene. The data represent the mean values ± SD of three independent experiments. The statistical differences between the untreated (control) and the treated (stimulated) group were calculated by one-way ANOVA analysis followed by Dunnett’s multiple comparisons post-test (* *p* < 0.05; ** *p* < 0.01, and *** *p* < 0.001).

Based on the results that show the anti-migratory potential of ASA, GEN, and the ASA+GEN combination, we further assessed the impact of these compounds (after a 24 h treatment) on the RNA expression of two matrix metalloproteinases (MMP-2 and MMP-9), markers of cancer cells’ invasion and metastasis ability, by performing an RT-qPCR analysis. The results from Figure 9 highlight that both ASA and GEN reduced the expressions of MMP-2 and MMP-9 in these cancer cells. However, the inhibition was stronger in the case of ASA-treated cells (2.5 mM) compared to GEN-treated ones (25 µM). The highest decrease in MMP-2 and MMP-9 mRNA expressions was obtained following the HCT-116 cells’ treatment with the ASA 2.5 mM + GEN 25 µM combination. 

### 3.6. ASA+GEN Combination Inhibits Angiogenesis In Ovo

**Figure 10:**
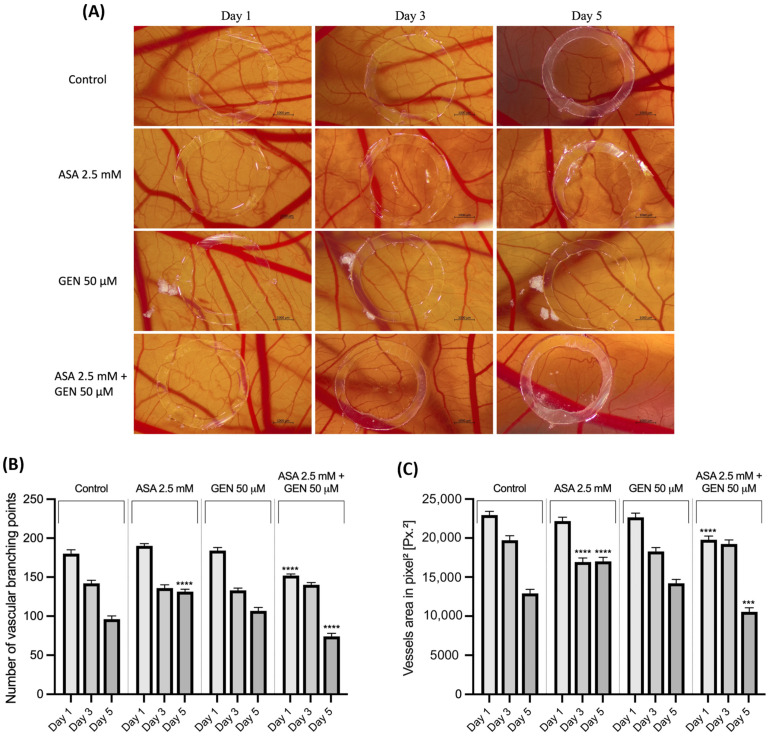
(**A**) Representative stereomicroscopic images of ASA (2.5 mM), GEN (50 μM), and the ASA (2.5 mM) + GEN (50 μM) combination effects on angiogenesis in the normal chick chorioallantoic membrane acquired on days 1, 3, and 5 of the experiment compared with the control group that received water (scale bars—1000 μm). (**B**) Quantitative evaluation of the number of vascular branching. (**C**) Quantitative measurement of the overall vessel area. The quantitative analysis was performed with IKOSA Prism AI Cam Assay (V3.1.0) for control, ASA, GEN, and ASA+GEN from day 1 to day 5. The statistical differences between the untreated (control) and the treated (stimulated) group were calculated by one-way ANOVA analysis followed by Dunnett’s multiple comparisons post-test (*** *p* < 0.001 and **** *p* < 0.0001).

Angiogenesis plays a pivotal role in cancer progression, including in CRC, so targeting this process represents a promising treatment approach [26]. To this end, we evaluated the impact of ASA—2.5 mM, GEN—50 μM, and the ASA 2.5 mM + GEN 50 μM combination on angiogenesis, using the chick embryo chorioallantoic membrane model. Changes in the vascular structures were monitored over a 5-day period. ASA treatment did not trigger significant alterations on angiogenesis during early germination, up to day 3, with the vessel’s aspect resembling the control group treated with water. Between day 3 and day 5, a subtle reduction in blood vessel diameter and vascular branching was noted. After administration of GEN during the heightened angiogenic phase, it was established that the compound preserved the normal functionality of the membrane’s vascular model, consistent with the observations noted in the control group. This preservation was characterized by a typical and progressive vascular branching pattern, particularly evident in the first three days of embryogenesis. On the fifth day of embryogenesis, a discernible decrease in the formation of vascular capillaries was noted, along with a reduction in vascular ramifications. However, administration of the ASA+GEN combination resulted in a marked modification of the vascular network. Notable changes included a reduction in vascular density and the number of capillaries within the vascular ring. It is noteworthy that during the experiments, no signs of vascular irritation, like hemorrhage (H), lysis (L), or vascular coagulation (C), were observed in any of the tested samples (Figure 10A). 

Following rigorous quantitative analysis, a notable decline in vascular branching was observed on the fifth day across all examined samples relative to the control. Notably, the most pronounced reduction in vascular branching points was documented in the cohort treated with a combination of ASA (2.5 mM) and GEN (50 μM) (Figure 10B). A parallel trend was evident in the total area of vessels, wherein a substantial decrease was noted specifically in the group treated with the association of the two compounds (Figure 10C). 

## 4. Discussion

The fight against cancer demands a relentless effort, mainly due to the fierceness and the perpetual resources of cancer cells to beat off chemotherapeutic treatments. Although great progress was made on research concerning the fundamental molecular mechanisms of cancer, monotherapy with chemotherapeutic agents continues to be the elective therapeutic choice for cancer treatment. Nonetheless, the severe side effects related to this type of therapy led to the embracement of chemoprevention as a promising measure to mitigate cancer incidence and to inhibit, prevent, or delay the tumoral process [27]. Chemoprevention implies a long-term administration of natural or synthetic drugs, so the safety profile of these compounds represents a primary concern when analyzing potential chemopreventive candidates [28]. In addition to chemoprevention, in recent years, different strategies were explored to remedy the drawbacks of anticancer therapy, such as drug resistance, lack of selectivity against cancer cells, severe side effects, and high costs, and one such approach can be considered the combination therapy. Moreover, the use of a combination of a repurposed drug (an FDA-approved agent for the treatment of other pathology) and other therapeutic compounds (of natural origin) offers benefits from both a therapeutical point of view, by enhancing the effectiveness of the treatment (e.g., via an additive or synergistic effect), and from an economical point of view, by decreasing the overall costs of the research [20]. Since both GEN and ASA were investigated as single agents for potential chemopreventive effects in CRC, we decided to analyze them as a combination (possible co-administration to CRC-diagnosed patients, mainly for post-menopausal women) to verify their impact on colorectal cancer cells. Moreover, it was suggested that ASA and flavonoids act as anticancer agents via a common mechanism represented by their metabolites, i.e., hydroxybenzoic acids [29].

In light of all these facts, the current study aimed to describe an in vitro evaluation of a potential chemopreventive alternative for colorectal cancer, i.e., the combination of aspirin (ASA—a repurposed drug) and genistein (GEN—a natural compound). Under the existing experimental conditions, the ASA+GEN combination showed a cytotoxic effect against colorectal cancer cells (HCT-116), characterized by a decline in cells’ viability percentage (Figure 1), notable morphological changes (round-shaped cells floating in the medium and reduced confluence) (Figure 3), reorganization of actin cytoskeleton and nuclear modifications (nuclear fragmentation and chromatin condensation) (Figure 4, Figure 5 and Figure 6), inhibition of tumor cells migratory capacity (Figure 7 and Figure 8), and reduction in MMP-2 and MMP-9 mRNA expressions (Figure 9), as well as an antiangiogenic effect by reducing the vascularization in the CAM model (Figure 10).

The in vitro assessment conducted in this study was selected based on the fact that the in vitro assays using cell lines represent the pipeline for the investigation of prospective cancer chemopreventive agents by evaluating their cytotoxicity in terms of cell death capacity, migratory potential, invasive capacity, and other underlying molecular mechanisms responsible for chemopreventive efficacy [27].

A point of interest of the present study was represented by the evaluation of genistein, an isoflavone found in soy-based foods and legumes that was intensively investigated as a chemopreventive candidate, in terms of cytotoxicity in HCT-116 cells. Our data showed a concentration-dependent (10–75 µM) reduction in HCT-116 cells’ viability (Figure 1), significant alterations of cellular morphology, a slight inhibitory effect on HCT-116 cells’ migratory capacity, and the reorganization of actin cytoskeleton and nuclear changes. These results are supported by other studies that demonstrated the anticancer effects of genistein in different colon cancer cells exerting multiple mechanisms of action, as follows: (i) in HT-29 and SW620 cells, genistein decreased cells’ viability and increased oxidative stress and inflammation [30]; (ii) in HT29 cells, genistein suppressed cells’ migration and invasion by inducing demethylation and recovering the activity of WNT inhibitory factor 1 (WIF1), a tumor suppressor [15]; (iii) in SW480 and SW620 cells, genistein triggered a dose-dependent antiproliferative effect and inhibited cells’ viability via apoptosis [14]; (iv) in HCT-116 cells, genistein induced apoptosis and suppression of cells’ proliferation by interfering with miR-95, Akt and SGK1 signaling transduction pathways [12], (v) in SW480, genistein treatment caused cell cycle arrest, inhibition of cells’ proliferation, and histone acetylation [31]; and (vi) in HCT-116 and LoVo cells, genistein induced the mitochondrial pathway of apoptosis by suppressing phosphorylation of Akt [32], etc. The anticancer potential of genistein was also proved in other preclinical models of breast cancer, gastric cancer, hepatocellular carcinoma, and salivary adenoid cystic carcinoma [27]. The main advantage of genistein is the low/non-toxic profile [14,33].

The chemopreventive properties of aspirin have been issued from different epidemiological studies since the 1980s, with their study being continued in multiple randomized controlled trials and meta-analyses that showed the impact of long-term use of aspirin (>10 years, low dose of aspirin) in declining the incidence and mortality of CRC and other types of cancer such as breast cancer, trachea, bronchus and lung cancers; nevertheless, the outcomes are debatable [20,28,34].

A second point of interest analyzed in the present study consisted of the investigation of ASA in different concentrations (1, 2.5, 5, and 7.5 mM, for 72 h) and its impact on human colorectal cancer cells (HCT-116), looking at cell viability percentage, cellular morphology changes, migratory capacity, cytoskeleton and nuclear alterations, and the expression of matrix metalloproteinases -2 and -9. The tested concentrations were selected after a review of the literature, which indicated that aspirin 1–10 mM proved to be cytotoxic in vitro against colon cancer cells, and, in addition, these concentrations were within the range of serum levels attained after pharmacological doses of aspirin [35]. Our results showed a dose-dependent cytotoxic effect defined by a significant decrease in HCT-116 cells’ viability percentage (Figure 1), cellular morphological changes (round cells, reduced confluence) (Figure 2), inhibition of the HCT-116 cells’ migratory potential (Figure 6), and apoptotic-like features (spherical cells, actin cytoskeleton reorganization, and nuclear alterations) (Figure 3). Similar effects were described by Pathi et al., who observed an inhibitory effect on colon cancer cells’ (SW480, RKO, HT-29, and HCT-116) growth, downregulation of Sp-related genes (key genes for cell proliferation, angiogenesis, and cell survival), and a caspase-dependent proteolysis of Sp1, Sp3, and Sp4 [35]. Another study conducted on human colon cancer cells—HCT-116 and HT29—demonstrated that aspirin (0–10 mM, for 72 h) triggered a dose-dependent decline in cells’ proliferation by inducing apoptosis and cell cycle G1 phase arrest [36]. Jung and coworkers proved that aspirin acted as an inhibitor of proliferation and an inducer of cellular senescence in SW620 and HCT-116 colorectal cells via activation of the SIRT1 and AMPK pathways [37]. The anticancer potential of aspirin was also demonstrated in preclinical models of hepatocellular carcinoma [38], breast cancer [39,40], and lung cancer [40].

A critical feature of any chemopreventive candidate is a low toxicity profile, with chemoprevention requiring prolonged use of the drug. Since the primary side effect of aspirin is gastrointestinal bleeding upon long-term administration, several strategies were explored to reduce aspirin side effects while retaining its efficacy, and one such approach would be the use of lower doses in combination with other agents. In recent years, different combinations of aspirin with both synthetic and natural compounds were tested, and the resulting findings were promising in terms of preclinical cancer prevention efficacy [28]. Natural compounds such as dietary phytochemicals (resveratrol, genistein, curcumin, etc.) are considered reliable candidates for their application in combination with chemotherapeutic drugs as a chemoprevention strategy, owing to their pleiotropic mechanism of action, and their non-toxic profile [28]. 

Based on the results described above related to ASA and GEN effects in HCT-116 cells and aiming to mitigate aspirin’s risk for developing side effects, our main hypothesis was to test the potential interaction between ASA and GEN in inducing cytotoxicity in human colorectal cells. The experiments performed confirmed our hypothesis, with significant effects being observed (reduced HCT-116 cells’ viability percentage, morphological alterations, inhibition of migratory potential, actin cytoskeleton remodeling and nuclear damage, and increased percentage of apoptotic nuclei) at the lowest concentration of ASA that decreased cells’ viability—2.5 mM—and the lowest concentration of GEN—10 µM—effects that were more pronounced compared to the ones described for GEN.

In the same vein, a recent study evidenced that the co-administration of aspirin with 5-fluorouracil (5-FU) and cisplatin augments the chemosensitivity of colorectal cancer cells (CT26 and HCT-116) [19]. A synergistic effect against colorectal cancer cells (HCT-116 and HT29) was observed using the combination of ASA and anti-Fas, a member of the tumor necrosis factor receptor family with a proapoptotic effect [41]. Previous studies proved the synergistic antitumor activity of aspirin when used in combination with 5-fluorouracil in hepatocellular carcinoma cells [42] and colorectal cancer cells [43], and in combination with erlotinib in preclinical non-small cell lung models [44].

Considering that both ASA and GEN, as well as the ASA+GEN combination, exerted a significant inhibition on the migration of HCT-116 cells, the study further investigated whether the underlying mechanism is related to the compounds’ ability to modulate the expression of two matrix metalloproteinases (MMPs), namely MMP-2 and MMP-9, key markers for cells’ invasion potential. Cancer invasion and metastasis are complex processes that start with the proteolytic degradation of the extracellular matrix (ECM) around the tumor, which subsequently eases the migration of malignant cells through ECM into surrounding tissues. MMPs such as the gelatinases MMP-2 and MMP-9 have a particular contribution to cancer invasion and metastases, with their overexpression being reported in numerous neoplasms, including CRC [45,46]. The experiment was performed using the concentrations at which the highest inhibition in the cells’ migratory ability was obtained—i.e., ASA 2.5 mM, GEN 25 µM, and ASA 2.5 mM + GEN 25 µM. The results indicated that the individual treatments with ASA and GEN reduced the mRNA expression of both MMP-2 and MMP-9, data that support previous reports. For instance, an early study revealed that ASA (0.3, 0.9, and 2.7 mM) inhibited the activity of MMP-2 (to 54%, 31%, and 20%) in SK-Hep-1 liver adenocarcinoma cells in a concentration-dependent manner, after 48 h of treatment [47]. A more recent study showed that ASA suppressed the invasion of prostate cancer cells by inhibiting MMP-9 activity [48]. Similar results were also obtained in the case of GEN (10, 20, and 60 µM) which dose-dependently reduced the expression of both MMP-2 and MMP-9 in HT-29 colorectal cancer cells after 72 h of treatment [15]. The results obtained in this work suggest that a higher inhibition in MMP-2 and MMP-9 expression was obtained following the HCT-116 cells’ combined treatment with ASA 2.5 mM + GEN 25 µM, compared to the individual treatments. 

Angiogenesis is known as one of the hallmarks of cancer and represents an essential step in cancer progression [49] and considering the antiangiogenic effect of both GEN [10] and ASA [50], the present study assessed the antiangiogenic potential of ASA+GEN combination in ovo, applying the CAM assay.

The chorioallantoic membrane (CAM) of chicken eggs emerges as a pivotal asset in the progression of medical investigations related to angiogenesis and cancer. Its paramount importance stems from its accessibility, optical clarity, and swift angiogenic responsiveness, rendering it an indispensable experimental model [51]. In the context of angiogenesis studies, the CAM serves as a dynamic arena for the observation and comprehension of the intricate processes governing blood vessel formation. Its transparent composition facilitates direct visualization of vascular changes, empowering researchers to scrutinize the effects of diverse factors on angiogenesis, encompassing both pro- and anti-angiogenic agents. Furthermore, the CAM’s facile experimental manipulation facilitates meticulous examinations into the molecular and cellular mechanisms orchestrating vascular development [52]. Several recent studies have indicated the potential role of aspirin in antitumor therapy through the suppression of angiogenesis [53,54,55]. Simultaneously, genistein has garnered attention due to its antitumor properties, partially attributed to its antiangiogenic effects [56]. 

Our findings revealed that ASA and GEN administration resulted in a subtle antiangiogenic effect. Notably, the most prominent effects, characterized by a reduction in capillary formation and a decrease in vascular branching, were observed with the combined application of both compounds. In the previously conducted study by Dai and colleagues, aspirin was assessed at concentrations ranging from 1 to 4 mmol/L on the chorioallantoic membrane to evaluate its effect on angiogenesis. The study results indicated that the highest tested concentration led to a decrease in blood vessel formation [53]. In a manner akin to this, Hu et al. assessed aspirin and its potential synergistic impact with erlotinib on the chorioallantoic membrane, and the results indicated a heightened suppression of blood vessel formation compared to the sole administration of aspirin [44]. Furthermore, the chorioallantoic membrane served as a platform in alternative investigations involving xenografts derived from C57BL/6 mice implanted with B16F10 melanoma cells. This approach was employed to evaluate the potential antitumoral properties of nanoparticles incorporating salicylic acid. The results of this study unveiled a notable antiangiogenic effect attributed to salicylic acid within the nanoparticles, leading to a reduction in capillary formation [57]. Importantly, these outcomes align with and substantiate the findings observed in this current study. 

Furthermore, prior investigations have explored the antiangiogenic potential of GEN on the chorioallantoic membrane. Specifically, one study focused on evaluating GEN at concentrations of 5 and 10 µg/mL (equivalent to GEN 18.5 and 37 µM), revealing a notable inhibition of angiogenesis, particularly at the 10 µg/mL dose, and when applied in a mixture of isoflavones [58]. Additionally, GEN has proven to be beneficial in preventing blood vessel permeability against lipopolysaccharides, thereby preventing the onset of acute inflammation on the chorioallantoic membrane [59].

## 5. Conclusions

In conclusion, the in vitro and in ovo results obtained indicate that the concurrent administration of the lowest dose of ASA (2.5 mM) + GEN (10–75 µM) induced a significant dose-dependent cytotoxic effect in human colorectal cancer cells (HCT-116) compared to the individual treatment with ASA or GEN, an effect defined by a decrease in cells’ viability percentage, modifications of cellular morphology (actin skeleton reorganization and nuclei impairment), reduced confluency, and apoptotic-like features. Moreover, an antimigratory effect was noted by suppressing cells’ migration and down-regulating MMP-2 and MMP-9 mRNA expressions. The ASA+GEN combination also exerted an antiangiogenic effect in the CAM model. These data represent a novel path for further exploration of the ASA+GEN combination’s therapeutic potential by elucidating their mechanism of action that impacts the carcinogenesis and angiogenesis processes in CRC. 

## Figures and Tables

**Table 1:** The oligonucleotides of the primers (GAPDH, MMP-2, MMP-9) used in the present study.

Name	Forward	Reverse
GADPH *	5′ AAG-GTG-AAG-GTC-GGA-GTC-AAC 3′	5′ GGG-GTC-ATT-GAT-GGC-AAC-AAT-A 3′
MMP-2	5′ ATG-ACA-GCT-GCA-CCA-CTG-AG 3′	5′ ATT-THT-TGC-CCA-GGA-AAG-TG 3′
MMP-9	5′ TTG-ACA-GCG-ACA-AGA-AGT-GG 3′	5′ GCC-ATT-CAC-GTC-GTC-CTT-AT 3′

* Housekeeping gene.

## Data Availability

The data presented in this study are available on request from the corresponding author.

## References

[1] Lepore Signorile M., Grossi V., Fasano C., Simone C. (2023). Colorectal Cancer Chemoprevention: A Dream Coming True?. Int. J. Mol. Sci..

[2] Kim S.H., Moon J.Y., Lim Y.J. (2022). Dietary Intervention for Preventing Colorectal Cancer: A Practical Guide for Physicians. J. Cancer Prev..

[3] The Global Cancer Observatory. https://gco.iarc.who.int/media/globocan/factsheets/populations/642-romania-fact-sheet.pdf.

[4] Baraibar I., Ros J., Saoudi N., Salvà F., García A., Castells M.R., Tabernero J., Élez E. (2023). Sex and gender perspectives in colorectal cancer. ESMO Open.

[5] Abancens M., Bustos V., Harvey H., McBryan J., Harvey B.J. (2020). Sexual Dimorphism in Colon Cancer. Front. Oncol..

[6] Das P.K., Saha J., Pillai S., Lam A.K., Gopalan V., Islam F. (2023). Implications of estrogen and its receptors in colorectal carcinoma. Cancer Med..

[7] Statista. https://www.statista.com/statistics/308333/dietary-supplement-usage-us-adults-by-gender/.

[8] Costea T., Hudiță A., Ciolac O.A., Gălățeanu B., Ginghină O., Costache M., Ganea C., Mocanu M.M. (2018). Chemoprevention of Colorectal Cancer by Dietary Compounds. Int. J. Mol. Sci..

[9] Delgado-Gonzalez P., Garza-Treviño E.N., de la Garza Kalife D.A., Quiroz Reyes A., Hernández-Tobías E.A. (2023). Bioactive Compounds of Dietary Origin and Their Influence on Colorectal Cancer as Chemoprevention. Life.

[10] Goh Y.X., Jalil J., Lam K.W., Husain K., Premakumar C.M. (2022). Genistein: A Review on its Anti-Inflammatory Properties. Front. Pharmacol..

[11] Liu X., Lan Y., Zhang L., Ye X., Shen Q., Mo G., Chen X. (2023). Genistein exerts anti-colorectal cancer actions: Clinical reports, computational and validated findings. Aging.

[12] Qin J., Chen J.X., Zhu Z., Teng J.A. (2015). Genistein inhibits human colorectal cancer growth and suppresses miR-95, Akt and SGK1. Cell Physiol. Biochem..

[13] Zhou P., Wang C., Hu Z., Chen W., Qi W., Li A. (2017). Genistein induces apoptosis of colon cancer cells by reversal of epithelial-to-mesenchymal via a Notch1/NF-κB/slug/E-cadherin pathway. BMC Cancer.

[14] Rendón J.P., Cañas A.I., Correa E., Bedoya-Betancur V., Osorio M., Castro C., Naranjo T.W. (2022). Evaluation of the Effects of Genistein In Vitro as a Chemopreventive Agent for Colorectal Cancer—Strategy to Improve Its Efficiency When Administered Orally. Molecules.

[15] Zhu J., Ren J., Tang L. (2018). Genistein inhibits invasion and migration of colon cancer cells by recovering WIF1 expression. Mol. Med. Rep..

[16] Guo C.G., Ma W., Drew D.A., Cao Y., Nguyen L.H., Joshi A.D., Ng K., Ogino S., Meyerhardt J.A., Song M. (2021). Aspirin Use and Risk of Colorectal Cancer Among Older Adults. JAMA Oncol..

[17] Perisetti A., Goyal H., Tharian B., Inamdar S., Mehta J.L. (2021). Aspirin for prevention of colorectal cancer in the elderly: Friend or foe?. Ann. Gastroenterol..

[18] Rashid G., Khan N.A., Elsori D., Rehman A., Tanzeelah, Ahmad H., Maryam H., Rais A., Usmani M.S., Babker A.M. (2023). Non-steroidal anti-inflammatory drugs and biomarkers: A new paradigm in colorectal cancer. Front. Med..

[19] Ying J., Zhou H., Wang Z., You Q., Chen J., Lu H., Zhang J. (2023). Aspirin increases chemosensitivity of colorectal cancer cells and inhibits the expression of toll-like receptor 4. Cancer Cell Int..

[20] Ai G., Dachineni R., Kumar D.R., Alfonso L.F., Marimuthu S., Bhat G.J. (2016). Aspirin inhibits glucose 6 phosphate dehydrogenase activity in HCT 116 cells through acetylation: Identification of aspirin-acetylated sites. Mol. Med. Rep..

[21] Davidson K.W., Barry M.J., Mangione C.M., Cabana M., Chelmow D., Coker T.R., Davis E.M., Donahue K.E., Jaén C.R., US Preventive Services Task Force (2022). Aspirin Use to Prevent Cardiovascular Disease: US Preventive Services Task Force Recommendation Statement. JAMA.

[22] Alzate-Yepes T., Pérez-Palacio L., Martínez E., Osorio M. (2023). Mechanisms of Action of Fruit and Vegetable Phytochemicals in Colorectal Cancer Prevention. Molecules.

[23] Pan P., Huang Y.W., Oshima K., Yearsley M., Zhang J., Yu J., Arnold M., Wang L.S. (2018). Could Aspirin and Diets High in Fiber Act Synergistically to Reduce the Risk of Colon Cancer in Humans?. Int. J. Mol. Sci..

[24] Lizeth G.R., Gustavo M.Q., Angie H.R., Wilson C.L., Andrés F.Y., Wilson C.G. (2022). New hybrid scaffolds based on ASA/genistein: Synthesis, cytotoxic effect, molecular docking, drug-likeness, and in silico ADME/Tox modeling. JAPS.

[25] Maghiari A.L., Coricovac D., Pinzaru I.A., Macașoi I.G., Marcovici I., Simu S., Navolan D., Dehelean C. (2020). High Concentrations of Aspartame Induce Pro-Angiogenic Effects in Ovo and Cytotoxic Effects in HT-29 Human Colorectal Carcinoma Cells. Nutrients.

[26] Hansen T.F., Qvortrup C., Pfeiffer P. (2021). Angiogenesis Inhibitors for Colorectal Cancer. A Review of the Clinical Data. Cancers.

[27] Mohan S.G., Swetha M., Keerthana C.K., Rayginia T.P., Anto R.J. (2022). Cancer Chemoprevention: A Strategic Approach Using Phytochemicals. Front. Pharmacol..

[28] Mohammed A., Fox J.T., Miller M.S. (2019). Cancer Chemoprevention: Preclinical In Vivo Alternate Dosing Strategies to Reduce Drug Toxicities. Toxicol. Sci..

[29] Sankaranarayanan R., Kumar D.R., Patel J., Bhat G.J. (2020). Do Aspirin and Flavonoids Prevent Cancer through a Common Mechanism Involving Hydroxybenzoic Acids?-The Metabolite Hypothesis. Molecules.

[30] Alorda-Clara M., Torrens-Mas M., Morla-Barcelo P.M., Roca P., Sastre-Serra J., Pons D.G., Oliver J. (2022). High Concentrations of Genistein Decrease Cell Viability Depending on Oxidative Stress and Inflammation in Colon Cancer Cell Lines. Int. J. Mol. Sci..

[31] Wang H., Li Q., Chen H. (2012). Genistein affects histone modifications on Dickkopf-related protein 1 (DKK1) gene in SW480 human colon cancer cell line. PLoS ONE.

[32] Qin J., Teng J., Zhu Z., Chen J., Huang W.J. (2016). Genistein induces activation of the mitochondrial apoptosis pathway by inhibiting phosphorylation of Akt in colorectal cancer cells. Pharm. Biol..

[33] Spagnuolo C., Russo G.L., Orhan I.E., Habtemariam S., Daglia M., Sureda A., Nabavi S.F., Devi K.P., Loizzo M.R., Tundis R. (2015). Genistein and cancer: Current status, challenges, and future directions. Adv. Nutr..

[34] Tsoi K.K.F., Ho J.M.W., Chan F.C.H., Sung J.J.Y. (2019). Long-term use of low-dose aspirin for cancer prevention: A 10-year population cohort study in Hong Kong. Int. J. Cancer.

[35] Pathi S., Jutooru I., Chadalapaka G., Nair V., Lee S.O., Safe S. (2012). Aspirin inhibits colon cancer cell and tumor growth and downregulates specificity protein (Sp) transcription factors. PLoS ONE.

[36] Luciani M.G., Campregher C., Gasche C. (2007). Aspirin blocks proliferation in colon cells by inducing a G1 arrest and apoptosis through activation of the checkpoint kinase ATM. Carcinogenesis.

[37] Jung Y.R., Kim E.J., Choi H.J., Park J.J., Kim H.S., Lee Y.J., Park M.J., Lee M. (2015). Aspirin Targets SIRT1 and AMPK to Induce Senescence of Colorectal Carcinoma Cells. Mol. Pharmacol..

[38] Shi T., Fujita K., Gong J., Nakahara M., Iwama H., Liu S., Yoneyama H., Morishita A., Nomura T., Tani J. (2020). Aspirin inhibits hepatocellular carcinoma cell proliferation in vitro and in vivo via inducing cell cycle arrest and apoptosis. Oncol. Rep..

[39] Maity G., De A., Das A., Banerjee S., Sarkar S., Banerjee S.K. (2015). Aspirin blocks growth of breast tumor cells and tumor-initiating cells and induces reprogramming factors of mesenchymal to epithelial transition. Lab. Investig..

[40] Li L., Hu M., Wang T., Chen H., Xu L. (2020). Repositioning Aspirin to Treat Lung and Breast Cancers and Overcome Acquired Resistance to Targeted Therapy. Front. Oncol..

[41] Szarynska M., Olejniczak-Keder A., Zubrzycki A., Wardowska A., Kmiec Z. (2021). Aspirin Exerts Synergistic Effect with Anti-Fas Stimulation against Colorectal Cancer Stem Cells In Vitro. Appl. Sci..

[42] Wang P., Shen Y., Zhao L. (2020). Chitosan nanoparticles loaded with aspirin and 5-fluororacil enable synergistic antitumour activity through the modulation of NF-κB/COX-2 signalling pathway. IET Nanobiotechnol..

[43] Susan M., Macasoi I., Pinzaru I., Dehelean C., Ilia I., Susan R., Ionita I. (2023). In Vitro Assessment of the Synergistic Effect of Aspirin and 5-Fluorouracil in Colorectal Adenocarcinoma Cells. Curr. Oncol..

[44] Hu X., Wu L.W., Weng X., Lin N.M., Zhang C. (2018). Synergistic antitumor activity of aspirin and erlotinib: Inhibition of p38 enhanced aspirin plus erlotinib-induced suppression of metastasis and promoted cancer cell apoptosis. Oncol. Lett..

[45] Araújo R.F., Lira G.A., Vilaça J.A., Guedes H.G., Leitão M.C., Lucena H.F., Ramos C.C. (2015). Prognostic and diagnostic implications of MMP-2, MMP-9, and VEGF-α expressions in colorectal cancer. Pathol. Res. Pract..

[46] Heslin M.J., Yan J., Johnson M.R., Weiss H., Diasio R.B., Urist M.M. (2001). Role of matrix metalloproteinases in colorectal carcinogenesis. Ann. Surg..

[47] Jiang M.C., Liao C.F., Lee P.H. (2001). Aspirin inhibits matrix metalloproteinase-2 activity, increases E-cadherin production, and inhibits in vitro invasion of tumor cells. Biochem. Biophys. Res. Commun..

[48] Shi C., Zhang N., Feng Y., Cao J., Chen X., Liu B. (2017). Aspirin Inhibits IKK-β-mediated Prostate Cancer Cell Invasion by Targeting Matrix Metalloproteinase-9 and Urokinase-Type Plasminogen Activator. Cell Physiol. Biochem..

[49] Mousa L., Salem M.E., Mikhail S. (2015). Biomarkers of Angiogenesis in Colorectal Cancer. Biomark. Cancer.

[50] Hoskin A.J., Holt A.K., Legge D.N., Collard T.J., Williams A.C., Vincent E.E. (2023). Aspirin and the metabolic hallmark of cancer: Novel therapeutic opportunities for colorectal cancer. Explor. Target. Antitumor Ther..

[51] Ribatti D., Ribatti D. (2010). Advantages and Limitations of Chorioallantoic Membrane in Comparison With Other Classical In Vivo Angiogenesis Assays. The Chick Embryo Chorioallantoic Membrane in the Study of Angiogenesis and Metastasis: The CAM Assay in the Study of Angiogenesis and Metastasis.

[52] Komatsu A., Higashi Y., Matsumoto K. (2019). Various CAM tumor models. Enzymes.

[53] Dai X., Yan J., Fu X., Pan Q., Sun D., Xu Y., Wang J., Nie L., Tong L., Shen A. (2017). Aspirin Inhibits Cancer Metastasis and Angiogenesis via Targeting Heparanase. Clin. Cancer Res..

[54] Zhao Q., Wang Z., Wang Z., Wu L., Zhang W. (2016). Aspirin may inhibit angiogenesis and induce autophagy by inhibiting mTOR signaling pathway in murine hepatocarcinoma and sarcoma models. Oncol. Lett..

[55] Maity G., Chakraborty J., Ghosh A., Haque I., Banerjee S., Banerjee S.K. (2019). Aspirin suppresses tumor cell-induced angiogenesis and their incongruity. J. Cell Commun. Signal.

[56] Yu X., Zhu J., Mi M., Chen W., Pan Q., Wei M. (2012). Anti-angiogenic genistein inhibits VEGF-induced endothelial cell activation by decreasing PTK activity and MAPK activation. Med. Oncol..

[57] Predoi M.C., Mîndrilă I., Buteică S.A., Purcaru Ș.O., Mihaiescu D.E., Mărginean O.M. (2020). Iron Oxide/Salicylic Acid Nanoparticles as Potential Therapy for B16F10 Melanoma Transplanted on the Chick Chorioallantoic Membrane. Processes.

[58] Su S.J., Yeh T.M., Chuang W.J., Ho C.L., Chang K.L., Cheng H.L., Liu H.S., Cheng H.L., Hsu P.Y., Chow N.H. (2005). The novel targets for anti-angiogenesis of genistein on human cancer cells. Biochem. Pharmacol..

[59] Ha Y.R., Ha H., Lee S.J. (2013). Protection of vessel permeability by genistein against lipopolysaccharide induced acute inflammation in a chick embryo chorioallantoic membrane model. Food Sci. Biotechnol..

